# Heterologous Immunization with Improved HIV-1 Subtype C Vaccines Elicit Autologous Tier 2 Neutralizing Antibodies with Rapid Viral Replication Control After SHIV Challenge

**DOI:** 10.3390/v17020277

**Published:** 2025-02-17

**Authors:** Gerald K. Chege, Rosamund E. Chapman, Alana T. Keyser, Craig H. Adams, Kealan Benn, Michiel T. van Diepen, Nicola Douglass, Bronwen Lambson, Tandile Hermanus, Penny L. Moore, Anna-Lise Williamson

**Affiliations:** 1 Primate Unit and Delft Animal Centre, Centre and Platform Office, South African Medical Research Council, Parrow Valley, Cape Town 7505, South Africa; 2 Division of Medical Virology, Department of Pathology, Faculty of Health Sciences, University of Cape Town, Cape Town 7925, South Africa; ros.chapman@uct.ac.za (R.E.C.); alana.keyser@uct.ac.za (A.T.K.); craig.adams@uct.ac.za (C.H.A.); kealanbenn@gmail.com (K.B.); mvand2009@hotmail.com (M.T.v.D.); niki.douglass@uct.ac.za (N.D.); anna-lise.williamson@uct.ac.za (A.-L.W.); 3 Institute of Infectious Disease and Molecular Medicine, Faculty of Health Sciences, University of Cape Town, Cape Town 7925, South Africa; 4 SA MRC Antibody Immunity Research Unit, School of Pathology, University of the Witwatersrand, Johannesburg 2000, South Africa; bronwenl@nicd.ac.za (B.L.); tandileh@nicd.ac.za (T.H.); pennym@nicd.ac.za (P.L.M.); 5 National Institute for Communicable Diseases, a Division of the National Health Laboratory Service, Johannesburg 2192, South Africa; 6 Centre for the AIDS Programme of Research in South Africa (CAPRISA), University of KwaZulu Natal, Durban 4001, South Africa

**Keywords:** immunogenicity, DNA vaccine, MVA vaccine, gp140 Env, SHIV, neutralization, Chinese rhesus macaques

## Abstract

We previously reported on HIV vaccines that elicited autologous Tier 2 neutralizing antibodies (nAbs) in rabbits. In the current study, we sought to establish a proof of concept that HIV vaccines using identical designs elicit Tier 2 nAbs in arhesus macaque (RM) model. DNA and MVA vaccines expressing SIV Gag and HIV-1 Env antigens were constructed, and in vitro expression was confirmed. A soluble envelope protein (gp140 Env) was expressed from a stable HEK293 cell line and purified using lectin affinity and size exclusion chromatography. The expression and secretion of SIV Gag and HIV-1 Env by the DNA and MVA vaccines was verified in vitro. Five RMs were inoculated with two DNA, followed by two MVA, and finally with two gp140 Env vaccines at weeks 0, 4, 8, 12, 20 and 28. Vaccine-induced T cell immunity was measured by IFN-γ ELISpot while nAbs were evaluated against MW965 (Tier 1A), 6644 (Tier 1B), autologous ZM109.5A and a closely-related ZM109.B4 (Tier 2) pseudovirions. Vaccinated RMs were challenged intrarectally with simian-human immunodeficiency virus (SHIV), four weeks after the final vaccination, as was an unvaccinated control group (*n* = 4). Following vaccination, all the animals developed moderate IFN-γ ELISpot responses after the DNA vaccinations which were boosted by the MVA vaccine. After the gp140 Env boost, all animals developed nAbs with peak median titres at 762 (MW965) and 263 (ZM109.5A). The vaccinated animals became infected after a similar number of challenges to the unvaccinated controls, and the resultant number of viral copies in the blood and the lymphoid tissues were similar. However, the duration of detectable viraemia in the vaccinated animals (median: 2 weeks) was shorter than the controls (median: 8.5 weeks). These data show that the vaccines elicited robust cellular and functional humoral immune responses that resulted in a quicker control of viraemia.

## 1. Introduction

A heterologous prime-boost strategy comprising DNA–MVA–Protein remains highly relevant in HIV vaccine research due to the induction of complementary immune responses and the potential of boosting the responses generated by the priming vaccine. A DNA vaccine primarily induces cellular immunity which can be boosted by the MVA vaccine while the protein vaccine component is crucial in generating antibody responses. Previously, our group developed two candidate HIV-1 vaccines (SAAVI DNA-C2 and SAAVI MVA-C) which were then tested in preclinical studies [1,2] and early-phase clinical trials (HVTN 073/SAAVI-102; 073E/SAAVI-102E and 086/SAAVI 103) [3,4,5]. Although these vaccines induced gp140 Env-binding antibodies and Tier 1A virus-neutralizing responses, no Tier 2 or broadly neutralizing antibody activity was observed [3,4]. In subsequent studies, the SAAVI MVA-C vaccine design was improved by the use of different promoters for the Gag and Env genes and replacing the native Gag gene with an in silico designed subtype C mosaic Gag antigen, resulting in the formation of Gag virus-like particles (VLPs) and much improvement in the induction of Gag-specific cellular responses [6,7].

Recent research efforts have maintained the inclusion of Gag while focusing on the modification of Env by sequence changes to improve the stability of Env trimers and their transport to the cell surface to generate better cellular and antibody responses to Tier 2 viruses [8,9]. Our recent research has made improvements to our vaccine candidates which have now been evaluated in the mouse and rabbit animal models [6,10,11,12,13,14]. Briefly, the improvements targeted the polyprotein, consisting of Gag, RT, Tat, Nef (*Grttn*), and Env, which were expressed by the DNA and MVA vectors in our previous SAAVI vaccines. The *Grttn* polyprotein was replaced with a subtype C mosaic Gag that is able to form virus-like particles and the Env was replaced with a modified Env which was derived from an individual in the South African CAPRISA acute infection cohort who developed broadly neutralizing antibodies [15]. The Env was truncated to gp150; the native leader sequence was replaced with the tissue plasminogen activator (TPA) leader peptide; the Furin cleavage site was replaced with a flexible Gly-rich linker sequence (GGGGSGGGGSG); a stabilizing I559P mutation was introduced into the gp41 region [10,11,12,13,14]. This Env, in conjunction with the mosaic Gag, was tested in rabbits in a DNA–MVA–Protein regimen, with two doses of each vaccine (DDMMPP) and was shown to induce high levels of Tier 1B and low levels of Tier 2 autologous neutralizing antibodies in rabbits [13]. Based on the DNA, MVA and gp140 protein vaccines used in these rabbit studies, we designed prototype vaccines to be tested in our Chinese rhesus macaque model with the SIV Gag and an HIV-1 subtype C Env corresponding to consensus sequence of the SHIVC109P7 [16], a virus derived from three in vivo serial passages of SHIVC109P4 [17] in Chinese rhesus macaques [16]. The Env sequences of SHIVC109P4 were originally derived from a southern African primary HIV-1 clade C isolate (ZM109) from an individual who was infected after heterosexual transmission [18,19,20]. The aim of the present study was, therefore, to test the DDMMPP vaccine regimen of these prototype vaccine formats containing SIV Gag and HIV-1 Env, in a non-human primate model, as a proof-of-concept study.

In the current study, the DNA and MVA vaccines expressing the SIV Gag and HIV-1 gp150, in combination with soluble gp140 proteins were constructed and tested in a group of five Chinese rhesus macaques using a similar heterologous vaccination (DDMMPP) regimen as that of the rabbit studies [13]. The vaccinated animals and a control group comprising of four unvaccinated macaques were challenged with SHIVC109P7. We showed that strong IFN-γ ELISpot (cellular) and moderate autologous Tier 2 antibody responses were elicited by the vaccine regimen. These responses did not confer protection against the SHIV challenge, but vaccination resulted in a shorter length of viraemia compared with the unvaccinated controls, suggesting that the vaccines enhanced the capacity the immune responses to control viraemia more rapidly.

## 2. Materials and Methods

### 2.1. Antibodies, Plasmids, Cell Lines, Media, and Reagents

Goat anti-HIV-1 gp160 (MRC ADP 72 408/5104), rabbit anti-HIV-1 p24 (Gag) (ARP Cat# 432; NIH AIDS Research and Reference Reagents Program, [now Biodefense and Emerging Infections Research Resources Repository; BEI Resources], Germantown, MD, USA), donkey anti-goat IgG Cy3 or FITC, and donkey anti-rabbit IgG Alexa 647 (ThermoFisher Scientific, Waltham, MA, USA) were used for immunofluorescence assays. Goat anti-human IgG-Cy3 (Fc-specific) antibody (Sigma-Aldrich, St. Louis, MO, USA) was used for a fluorescence activated cell sorting (FACS) assay. Goat anti-HIV-1 gp120 (BioRad, Hercules, CA, USA), goat anti-HIV-1 p24 (Gag) (BioRad, Hercules, CA, USA) and mouse monoclonal anti-goat/sheep IgG–AP GT34 (Sigma-Aldrich, St. Louis, MO, USA) were used for western blotting. Anti-HIV-1 Env human monoclonal antibodies (MAbs) PG9, PG16, PGT128, PGT145, CAP256-VRC26.08, VRC01, 10E8 and 447-52D were expressed in FreeStyle^TM^ 293F cells (ThermoFisher Scientific, Waltham, MA, USA) using the PEIMAX transfection reagent (Polysciences, Warrington, PA, USA). Monoclonal antibodies were purified from cell-free supernatants after 6 days using Protein-A affinity chromatography [21].

### 2.2. Design of DNA, MVA, and Protein Subunit Vaccines

The envelope sequence was derived from the clade C SHIV, SHIVC109P7, which had been adapted for replication in Chinese-origin rhesus macaques via a rapid three-step in vivo serial animal-to-animal passage [16]. The consensus envelope sequence of the SHIVC109P7 stock [16] was used in order to match the vaccines as closely as possible to the challenge virus. The envelope sequence was altered to improve expression and trimer formation as described previously: the native leader sequence was replaced with the TPA leader peptide, the Furin cleavage site was replaced with flexible linker (FL) consisting of 2 repeats of (GGGGS)_2_, and an isoleucine was mutated to proline (I559P) in the gp41 region [13]. The envelope was truncated to gp150 for the DNA and MVA vaccines and gp140 for the soluble protein subunit vaccine. The Gag sequence used in the DNA and MVA vaccines was derived from SIVmac239 (GenBank: AAA47632.1). For all constructs, any potential poxvirus termination signals (TTTTTNT) were removed from the coding sequence, and a poxvirus termination signal was added directly after the stop codon of the envelope gene.

The mammalian expression plasmid pTHPcapR [22] was used to construct DNA vaccines pMExT SHIV gp150 and pMEx SIV Gag expressing the SHIVC109P7 envelope and SIV Gag respectively. All DNA vaccines were synthesized by GenScript (Piscataway, NJ, USA).

A recombinant MVA co-expressing matching Env and Gag antigens was constructed as described by van Diepen et al., 2019 [13]. A transfer vector was created, containing an expression cassette between flanking sequences of the MVA G1L–I8R locus. The expression cassette contained eGFP under the control of the vaccinia virus (VACV) p7.5 promoter, K1L under the control of the VACV pSS promoter, gp150 under the control of the VACV mH5 promoter, and Gag under the control of the pLEO promoter. The transfer vector allowed for positive selection of integration into MVA via K1L selection in RK13 cells and identification of recombinant virus by eGFP expression.

A stable HEK293 cell line expressing SHIVC109P7 gp140 protein (soluble Env) was made as described previously [14]. Soluble Env was isolated from the media of serum-starved stable cell lines and purified using lectin affinity and size exclusion chromatography as described previously. To confirm isolation of gp140 Env, samples were separated on precast NativePAGE™ Novex^®^ 3–12% Bis-Tris Protein Gels (ThermoFisher Scientific, Waltham, MA, USA) stained with Bio-Safe Coomassie (Bio-Rad, Hercules, CA, USA).

### 2.3. Confirmation of Gag and Env Expression by DNA and MVA Vaccines

Transfections of HEK293T cells were performed in 4 Well Permanox^®^ Slides (Lab-Tek^®^, Brendale, Australia) for immunofluorescent staining using 1 µg of plasmid DNA (0.5 µg Env + 0.5 µg Gag) and 3 µL X-tremeGENE (Roche, Basel, Switzerland). HEK293T cells were infected at a MOI of 0.5 with recombinant MVA. Western blotting and immunofluorescent staining of cells was carried out as described by van Diepen et al., 2019 [13].

### 2.4. VLP Isolation and Characterisation

In order to visualize VLPs on EM grids, HEK293T cells were transfected in T175 flasks using 60 µg of plasmid DNA (30 µg Env + 30 µg Gag) and 180 µL PEI or infected at a MOI of 1.0 with MVA. After 72 h, VLPs were filtered through a 0.2 µm filter and then pelleted through a 5 mL 12% OptiPrep (in TBS) cushion. The samples were centrifuged at 50,000× *g*, 4 °C, with no breaks, for 1 h. Pellets containing VLPs were reconstituted in 100 µL TBS + 20% glycerol. Freshly activated carbon grids were coated with VLP preparations, stained with uranyl acetate and imaged on a FEI/Tecnai T20 TEM (ThermoFisher (formerly FEI), Eindhoven, The Netherlands.

### 2.5. SHIVC109P7 Challenge Virus Stock

SHIVC109P7 challenge virus stock was generated from the original SHIVC109P4 stock as described [16]. In brief, SHIVC109P4 seed stock donated by Cecilia Cheng-Mayer was adapted for replication in Chinese-origin RMs via three intravenous, serial passages. The SHIVC109P7 virus stock used for challenge in this study was derived from the final passage. The virus was quantified by enzyme-linked immunosorbent assay (ELISA) for SIV p27 antigen (Advanced Bioscience Laboratories, ABL, Rockville, MD, USA) and the infectivity was measured in TZM-bl cells in a previous study [16]. In addition, the viral stock was titrated in vivo in RMs and a 1:100 dilution corresponding to 6000 TCID_50_ per inoculation was established as a suitable low dosage for intrarectal challenges [16] in the current vaccine study.

### 2.6. Animal Immunizations and SHIV Challenges

Nine rhesus macaques (*Macaca mulatta*) of Chinese origin (RMs) were allocated to the experimental (A; *n* = 5) and the control (B; *n* = 4) groups. All animals were seronegative for SIV, simian retrovirus type D, simian T-cell leukemia virus, and monkey B virus. The animals in the experimental group received two DNA vaccine primes (DD), four weeks apart (1 mg DNA per monkey), two MVA vaccine boosts (MM), four weeks apart (10^8^ pfu rMVA per monkey), and finally two gp140 Env protein boosts (PP), eight weeks apart (200 µg gp140 Env per monkey formulated 1:1 (*v*/*v*), with AlhydroGel adjuvant (InvivoGen, San Diego, CA, USA). The vaccines were administered via the intramuscular route into the quadriceps muscles. Animals in the control group were left unvaccinated. Starting at Week 32, the animals in both groups were repeatedly challenged intrarectally (i.r.) with SHIVC109P7 (1 mL; 6000 TCID_50_ per animal per challenge), until infection was confirmed. Repeated low-dose rectal inoculations of rhesus macaques with SIV or SHIV is thought to best recapitulate natural mucosal infection of humans with HIV-1 and therefore, it is an accepted challenge method for vaccine efficacy evaluations [23]. Week 32 corresponded to 4 weeks after the last gp140 Env protein vaccine. Peripheral blood was obtained by venipuncture of the femoral vein and aseptically collected into vacutainer blood tubes for isolation of peripheral blood mononuclear cells (PBMCs) at various timepoints. At the experimental endpoint, spleen and mucosa-associated lymph nodes (inguinal, mesenteric) were harvested for isolation of splenocytes and lymphoid mononuclear cells (LMNCs), respectively.

The animals were housed at the South African Medical Research Council’s (SAMRC) animal facility in Cape Town, and they were maintained in accordance with the South African national guidelines for Use of Animals for Scientific Purposes (SANS Code 10386) which is compliant with the EU Directive 2010/63/EU for animal experiments. In addition, the ARRIVE guidelines were used in the implementation of the studies. The experimental protocol was independently reviewed and approved by the Animal Ethics Committees of the University of Cape Town (AEC 014/044) and the SAMRC (ECRA 06/14).

### 2.7. Isolation of PBMCs, Splenocytes and LMNCs

PBMCs were isolated from heparinized whole peripheral blood by standard Ficoll gradient centrifugation while splenocytes and LMNCs were isolated from spleens and lymph nodes, respectively, as previously described [2]. PBMCs that were not used in IFN-γ ELISpot assays, splenocytes and LMNCs obtained at experimental endpoint were resuspended in fetal bovine serum (FBS, Gibco^TM^, Invitrogen, Carlsbad, CA, USA) containing 10% dimethyl sulfoxide (DMSO; Sigma-Aldrich, St. Louis, MO, USA) and cryopreserved in liquid nitrogen until use in viral load assays.

### 2.8. IFN-γ ELISpot Assay

Freshly isolated PBMCs were used in a standard IFN-γ ELISpot assay, as previously described [16,24]. Briefly, the PBMCs were incubated at 37 °C for 22–24 h in triplicates in the presence or absence of either SIVmac239 Gag (ARP Cat# 6204) or HIV-1 Consensus Subtype C Env (ARP Cat#: 12634) peptide pools at 1 µg/mL each. Pre-mixed peptide sets were procured from BEI Resources (Germantown, MD, USA). The spot counts were normalized to spot forming units (SFU) per million PBMCs after subtracting the background (in absence of peptide pools) and the cut-off value for a positive response was set at 30 SFU/million PBMCs based on pre-vaccination peptide reactivity. A response below the cut-off value was assigned a magnitude of zero. The results are given as cumulative SFU/million PBMCs for both peptide pools.

### 2.9. Binding Antibody ELISA

Gp140 Env-specific IgG antibody responses were measured in a standard ELISA in 4-fold serial dilutions starting at a serum dilution of 1:20 as previously described [2]. Maxisorb microtitr ELISA plates (Nunc^TM^, ThermoFisher Scientific, Waltham, MA, USA) were coated with soluble gp140 Env trimers [14] at 10 ng/well. Gp140 Env-specific binding antibodies were measured at pre-vaccination (baseline) and the following post-vaccination timepoints: DD (2 weeks after receiving the 2nd DNA), DDMM (1 week after the 2nd MVA), DDMMP (2 weeks after the 1st gp140 Env), DDMMPP (2 weeks after the 2nd gp140 Env), pre-challenge (at the start of the SHIV challenges which coincided with 4 weeks after the 2nd gp140 Env booster), and post-challenge (experimental endpoint which coincided with 21 weeks after the 1st i.r. SHIV challenge). Data are presented as endpoint antibody titr which were defined as the reciprocal of the highest serum dilution with an absorbance that was equal or greater than two times the mean absorbance of the negative control. A titre <20 (based on baseline and unvaccinated controls) was considered a negative antibody response.

### 2.10. HIV-1 Neutralizing Antibody Assay

HIV-1 neutralizing antibodies were measured against HIV-1 Env-pseudotyped viruses from Tier 1 (MW965.26, 6644.v2.c33 and ZM109.B4) and Tier 2 (ZM109.5A N276D G394S) viruses using the TZM-bl assay as previously described [25]. ZM109.B4 pseudovirus was selected as it contains the HIV-1 envelope sequence used to construct SHIVC109F.PB4 [17]. There were 105 amino acid differences and 87.86% identity between the ZM109F.PB4 envelope sequence and the SHIVC109P7 amino acid sequence utilized in constructing the vaccines. SHIVC109P7 was derived from SHIVC109P4 which was classified as a Tier 2 virus [17]. Thus, ZM109.5A N276D G394S (ZM109.5A), which was specifically constructed for this study, with a matching sequence to the vaccine and the consensus Env sequence of the SHIVC109P7 was an autologous Tier 2 pseudovirus. Neutralizing antibodies were measured at baseline and at the following post-vaccination timepoints: DDMM, DDMMP, DDMMPP, and pre-challenge. Titres were calculated as the reciprocal of the highest plasma dilution resulting in a 50% reduction in relative luciferase units [RLU]. A titre <20 is the lowest level of detection for neutralization and titre >96 (mean background + standard deviation) was considered the cut-off value for positive nAb titres based on values for ZM109.5A neutralization responses where the background (baseline timepoints) was >20 (mean: 71; standard deviation: 25).

### 2.11. Measurement of Viral RNA Levels in the Peripheral Blood

Plasma viral RNA was isolated using QiaAmp viral RNA minikit (Qiagen, Valencia, CA, USA) and viral RNA levels were measured by quantitative reverse transcriptase PCR (qRT-PCR) for SIV gag sequences, as previously described [26]. qRT-PCR was conducted using TaqMan probe chemistry (TaqMan™ Gene Expression Master Mix; ThermoFisher Scientific, Waltham, MA, USA) on a QuantStudio™ 7 Flex Real-Time PCR System (Applied Biosystems, ThermoFisher Scientific, Waltham, MA, USA), as described previously [26]. The limit of detection was estimated at 16 RNA copies per mL. Data were expressed as the number of SIV DNA copies per mL of plasma.

### 2.12. Measurement of Viral RNA and Proviral DNA Levels in PBMC and Lymphoid Tissues

RNA and genomic DNA were extracted from ten million cryo-preserved PBMCs, splenocytes, and LMNCs using the QIAamp blood kit (Qiagen, Valencia, CA, USA) as previously described [16]. The viral RNA and proviral DNA levels were measured by qRT-PCR for SIV gag sequences, as described above. The qRT-PCR primers and probe amplified a 310 bp fragment of the SIV gag gene. The standard curve was generated by a 5-fold serial dilution from 50,000 copies of plasmid DNA down to 3.2 copies. Data were expressed as the number of SIV gag RNA or DNA copies per 10 million cells.

### 2.13. Envelope Sequencing

For sequence analysis, viral RNA was extracted using an RNA extraction kit (Qiagen, Valencia, CA, USA). This was followed by reverse transcription with SuperScript III reverse transcriptase (Invitrogen) and random hexamer primers (Amersham Pharmacia, Piscataway, NJ, USA). To perform single genome amplifications (SGAs), cDNA was titrated through nested PCR of the HIV-1 env gene using 9 replicates per dilution. The dilution yielding 2 out of 9 (less than 30%) positive reactions was selected to produce HIV-1 env single genome amplicons [20,27]. Sanger sequencing of these amplicons, as previously detailed [28], was conducted by the Central Analytical Facility at Stellenbosch University. The Sanger reads were assembled into contigs using CLC Sequence Viewer 6.7.1 software (CLC bio A/S, Aarhus, Denmark). Env sequences were assembled and aligned using CLC. Neighbour-joining trees were constructed using the Jukes–Cantor distance measure and 1000 bootstrap replicates.

### 2.14. Measurement of Absolute CD4^+^ T Cells

EDTA-anticoagulated whole blood was used to determine the CD4^+^ T cell counts in the peripheral blood using reagents from BD Biosciences (Franklin Lakes, NJ, USA) according to the manufacturer’s guidelines as previously described [16]. Data were acquired on the BD FACSCalibur using the BD Multiset software version 3.1x (BD Biosciences, Franklin Lakes, NJ, USA) and analyzed using FlowJo 9 (TreeStar, LaJolla, CA, USA). The results are shown as absolute CD4^+^ T cell counts per µL blood.

### 2.15. Data Analysis

Statistical analyses were performed using GraphPad Prism software version 10.0 (Boston, MA, USA). Statistical comparisons between magnitudes of cumulative ELISpot, antibody titers. and viral loads in the vaccinated and unvaccinated groups were performed using Mann–Whitney U test while the Gehan–Breslow–Wilcoxon and log-rank tests were used to assess the survival rates and hazard ratio respectively after SHIV challenges. All statistical tests were two-tailed and *p*-values less than 0.05 were considered significant.

## 3. Results

This section may be divided by subheadings. It should provide a concise and precise description of the experimental results, their interpretation, as well as the experimental conclusions that can be drawn.

### 3.1. Design, Construction and Production of Vaccines

**Figure 1:**
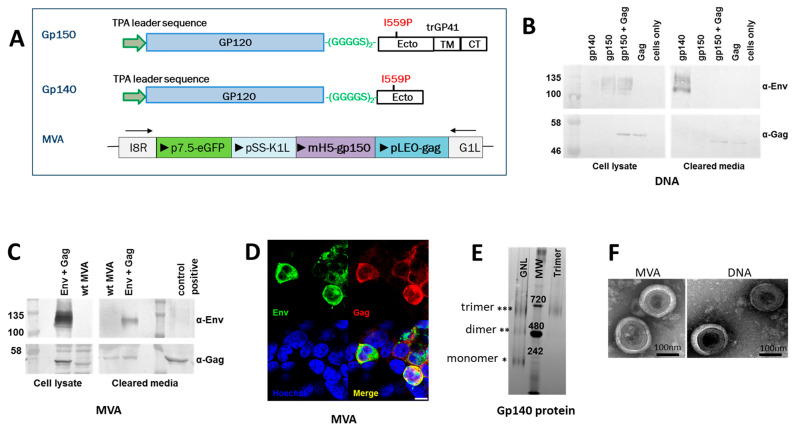
Design of vaccines utilized in this study. (**A**) Schematic representation of Gp150 Env. The gp160 sequence was truncated at amino acid residue 730 to generate gp150, the native signal peptide sequence was replaced with the human tissue plasminogen activator leader sequence (TPA), the Furin cleavage motif was replaced with a flexible linker (GGGGS)_2_, and an I559P mutation was introduced. The soluble protein, Gp140, was truncated at amino acid 653. Schematic representation of recombinant MVA vaccine; four genes (eGFP, K1L, gp150, and gag) were inserted between the MVA ORFs I8R and G1L; triangles indicate the direction of transcription from promoters p7.5 (eGFP), pSS (K1L), mH5 (gp150) and pLEO (gag). Western blot analysis of cell lysate or clarified media (secreted protein) from cells transfected with DNA vaccines (**B**) and infected with MVA vaccines (**C**). (**D**) Confocal microscopy of cells infected with MVA show that both Env (green, Cy3) and Gag (red, Alexa Fluor 647) were expressed. (**E**) Coomassie-stained Blue Native PAGE of soluble Env purified from a stable cell line by *Galanthus nivalis* lectin affinity chromatography (GNL) followed by size exclusion chromatography (Trimer). Molecular weight (MW) is indicated in kDa. (**F**) Negative stain EM analysis of SIV Gag VLPs expressed from cells infected with MVA or transfected with DNA vaccines. Resolution 0.52 nm (5.2 A/pixel).

The wild-type SIVmac239 Gag and an Env derived from SHIVC109P4 [17] were used to generate the DNA and MVA vaccines. SHIVC109P4 was derived from HIV-1 subtype C ZM109 isolate as previously described [17]. The Env sequence used to generate the vaccines was derived from the consensus sequence of SHIVC109P7 which was generated following serial passage of SHIVC109P4 in Chinese rhesus macaques [16]. The consensus sequence of SHIVC109P7 had 98.49–99.77% sequence identity to the other clones in the final passage and the amino acid differences ranged from 2–13. The DNA and MVA vaccines were designed to express a membrane-anchored gp150 (Env) with the aim that the co-expression with the SIV Gag would lead to the incorporation of HIV-1 Env into Gag virus-like particles (VLPs). Previous work by our group has shown that the presentation of Env on the surface of Gag VLPs leads to better neutralizing immune responses when compared to the HIV-1 envelope protein alone [13]. The envelope sequence was modified, as previously described [13], as shown in Figure 1A. The mammalian expression vector pTHPCapR, containing the porcine circovirus enhancer element, which has been shown to give increased antigen expression and immunogenicity [22], was utilized for the DNA vaccines expressing HIV-1 Env and SIV Gag [6,13]. The recombinant MVA vaccine contains the HIV-1 gp150 under the control of the VACV mH5 promoter and Gag under the control of the pLEO promoter. Both genes were inserted into a stable, intergenic region of the MVA genome, between the convergent, open reading frames I8R and G1L as shown in Figure 1A [13]. Soluble gp140 Env protein was purified using Galanthus nivalis lectin (GNL) affinity chromatography followed by size exclusion chromatography (Figure 1E).

### 3.2. Expression and Characterisation of HIV Envelope

Expression of Gag and Env in cells co-transfected with the DNA vaccines or infected with the MVA vaccines was confirmed by western blotting (Figure 1B,C) and confocal microscopy (Figure 1D). Gag and Env were also detected in the cell media by western blot analysis, confirming that the proteins were secreted (Figure 1B,C).

### 3.3. Characterisation of VLPs

Both the DNA and MVA vaccines produced virus-like particles, as shown by transmission electron microscopy (TEM) of transfected/infected cells (Figure 1F). No VLPs were seen for uninfected/untransfected cells or cells infected with parental MVA or LSDV, confirming that VLPs production was vaccine specific.

### 3.4. Experimental Design, Vaccinations, and Sampling

**Figure 2:**
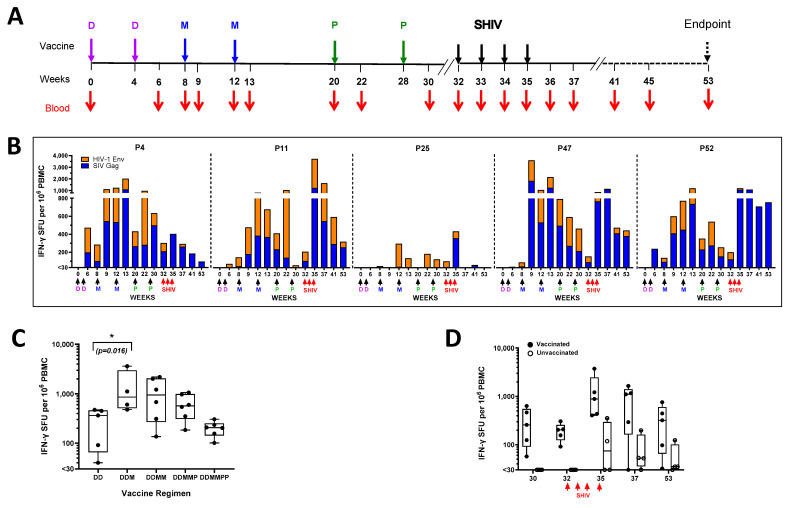
Animal experimentation and IFN-*γ* ELISpot responses. (**A**) Schematic diagram of the experimental design showing times of vaccinations, intrarectal SHIV challenges, and blood sampling. (**B**) Cumulative IFN-*γ* ELISpot responses of individual vaccinated animals at baseline (week 0) and various timepoints. Each graph represents an individual animal, with each bar representing cumulative IFN-*γ* ELISpot responses to SIV Gag, and HIV-1 Env peptide pools measured as spot forming units (SFU) per million PBMC. Magnitudes equal or less than 30 SFU/million PBMC were deemed as negative and assigned zero. Grouped IFN-*γ* ELISpot responses at key timepoints showing vaccinated (solid circles) and unvaccinated (open circles) prior to SHIV challenge (**C**) and after challenge (**D**). Each circle represents the cumulative (SIV Gag + HIV-1 Env) response of an individual animal. (DD: two weeks after two DNA vaccines; DDM: one week after two DNA and the first MVA vaccines; DDMM: one week after two DNA and two MVA vaccines; DDMMP: two weeks after two DNA, two MVA, and first protein vaccines; DDMMPP: two weeks after two DNA, two MVA, and two protein vaccines). D = DNA vaccine; M = MVA vaccine; P = protein vaccine; SHIV = SHIV challenge.

We designed the NHP experiment based on our rabbit experiments [13] which had shown optimal induction of antibody responses following vaccination with 2 DNA primes followed by 2 MVA boosts, given 4 weeks apart, followed by 2 gp140 Env protein, given 8 weeks apart (Figure 2A). We chose DNA and MVA dosages that were 4-fold and 10-fold lower, respectively, compared with our original SAAVI vaccines due to observed improvement in antigen expression and immunogenicity [12,13,22]. The protein vaccine was formulated in AlhydroGel, an alum-based adjuvant, as our rabbit studies had shown induction of higher antibody titres compared with AddaVax, an oil-in-water emulsion like MF59 adjuvant [14]. To assess vaccine efficacy, we then introduced a control group at this point and challenged both groups with a SHIV stock which we had previously titrated in vivo [16], at an intra-rectal dose of 6000 TCID_50_, weekly.

### 3.5. IFN-γ ELISpot Responses

We measured T cell responses to the vaccine immunogens using peptide pools of SIV Gag and HIV-1 subtype C consensus Env (NIH AIDS Reagent Program) in an IFN-γ ELISpot assay at baseline and several time points post vaccination (Figure 2A). Two weeks after two DNA vaccine primes (DD), 4 out of 5 vaccinated animals generated vaccine responses with a cumulative median of 228 (range: 433) SFU per million PBMC (Figure 2B) and by week 8, before the animals received the first MVA booster vaccination, all animals had developed responses (median: 147; range: 235 SFU per million PBMC). As expected, the cumulative magnitudes of responses increased significantly (*p* = 0.029) after the first MVA boost (DDM) in 4 of 5 animals (median: 860; range: 3144 SFU per million PBMC), (Figure 2C) while there was a delayed boosting outcome in 1 animal. Increases in magnitudes were observed in 3 of the 5 animals following booster immunization with the second MVA (DDMM), although the cumulative median response continued to decline even further despite the gp140 Env boosters. However, all animals had responses at the time repeated i.r. SHIV challenges commenced (median: 203; range: 203 SFU/million PBMC), resulting in the restoration of high-magnitude responses. As expected, the unvaccinated controls mounted only low to moderate IFN-γ ELISpot responses after commencing i.r. SHIV challenges with a median of 74 SFU/million PBMC (range: 323 SFU/million PBMC) at week 35 after 2–3 challenges (Figure 2D; Supplementary Figure S1).

Overall, we observed induction of modest IFN-γ ELISpot responses following two DNA vaccinations, which were further boosted by the first MVA. The magnitudes declined steadily thereafter until the time of SHIV challenges, after which they were again re-boosted substantially in the majority of animals.

### 3.6. Antibody Responses

Next, we evaluated the vaccine-generated antibody responses by investigating the gp140 Env-binding IgG titers using an ELISA method and nAb responses against 4 HIV-1 Env-pseudotyped viruses in TZM-bl assays. For both assays, testing was performed at time points following at DDMM, DDMMP, DDMMPP, and pre-challenge (4 weeks after the second gp140 Env) as well as pre-vaccination (baseline). In addition, the binding antibody titers were measured at DD and post-challenge timepoints.

**Figure 3:**
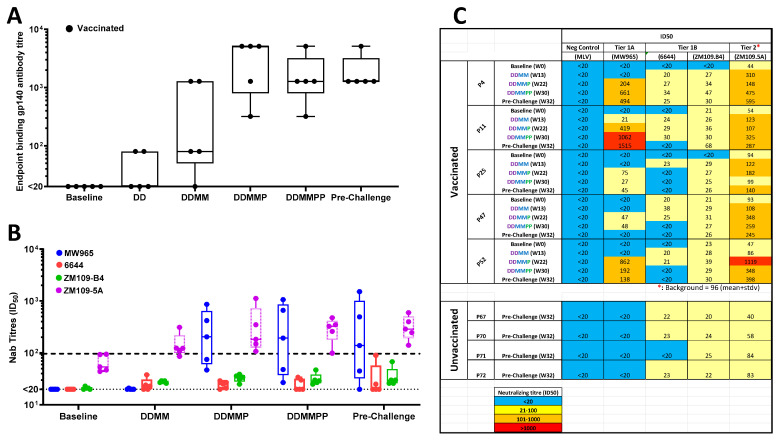
Antibody responses following vaccinations. (**A**) A graph showing Gp140 Env-binding antibody titres at key timepoints measured by ELISA. Each circle represents an endpoint titre of an individual animal at the specified timepoint. (**B**) A graph showing neutralizing antibody titres to MW965.26, 6644, ZM109.B4, and ZM109.5A pseudovirions at the specified timepoints. Each circle (blue, red, green, or purple) on the whisker and box bars represents the antibody titre that caused a 50% reduction in relative luciferase units, and the horizontal lines within the bars represent the median titres for the timepoints. The dotted lines at titre <20 and 96 show the lowest level of detection and the cut-off value for positive nAb titres respectively. (**C**) A table showing neutralizing antibody titres to MW965, 6644, ZM109.B4, and ZM109.5A at the specified timepoints. A titre <20 was considered negative for neutralization. ID_50_ values are shown with resistance being >20 (blue) and increasing sensitivity of 20–100 (yellow), 101–1000 (orange), and >1000 (red). (*: denotes a background value of 96 representing a mean of 71 plus a standard deviation of 25).

The ELISA data revealed that while both the DNA and MVA vaccines generated modest titers in 2 of 5 (median: 80) and 4 of 5 (median: 680) vaccinated animals, respectively, the first gp140 Env protein boost generated robust antibody titers (median: 5120; range: 320–5120) (Figure 3A). The second gp140 Env boost did not increase the antibody titers in the majority of animals, but the same level of responses was maintained until the time of SHIV challenges (Figure 3A). The high levels of antibodies were maintained post-challenge (median: 1280; range: 1280–5120) which was slightly surpassed, but not significantly (*p* = 0.4), by titers in the unvaccinated animals post-challenge (median: 3200; range: 1280–20480) (Supplementary Figure S2A).

Moderate neutralization activity was observed against the MW965, a Tier 1A virus, in the majority of animals with a median titer of 204 (range: 27–862) at DDMMP and reaching a high of 1062 (median: 192) at DDMMPP (Figure 3B,C). Surprisingly, neutralization activities against 6644, a Tier 1B pseudovirus, and against ZM109.B4, a Tier 2 pseudovirus containing the Env sequences derived from the Zambian isolate from which the initial SHIVC109F.PB4 was constructed, were within the background (titer of 96). The autologous neutralization titers to ZM109.5A (a pseudovirus with an Env sequence matching the vaccines) were higher than the background, reaching a median peak of 325 (range: 99–475) at DDMMPP. Two of five animals (P52 and P4) reached peak titers of 1119 and 595 against ZM109.5A at DDMMP and re-challenge timepoints, respectively. It was noted that 1 of the 5 vaccinated animals (P25) was a poor responder, developing no nAbs or only modest responses to some pseudoviruses. Overall, the trend of nAbs levels increased steadily with each vaccination and reaching the highest median at DDMMPP with all the animals showing detectable autologous nAb to ZM109.5A at the time of SHIV challenge (Supplementary Figure S2B). As expected, pre- and post-challenge samples from the unvaccinated control animals showed no or background (titer of <96) neutralization activities, with no differences to those observed for the vaccinated group against 6644 and ZM109.B4 viruses. (Figure 3C).

There was no correlation between the levels of the binding gp140 Env antibody titers and nAb titers against MW965 or ZM109.5A at DDMMP, DDMMPP, or pre-challenge time points (Supplementary Figure S2C) which was also a surprising outcome.

### 3.7. SHIV Challenge Outcomes

**Figure 4:**
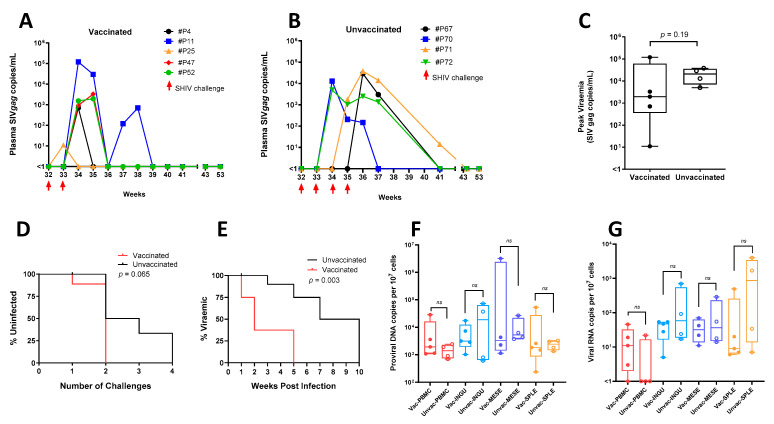
SIV Gag viral loads following repeated intrarectal SHIV challenges. SHIV plasma viral loads in the peripheral blood of vaccinated (**A**) and unvaccinated (**B**) macaques following intrarectal SHIV challenges. Each line graph represents an individual animal at pre-inoculation (Week 32) and post-inoculation with SHIV. Peak viral loads of vaccinated and unvaccinated macaques following SHIV challenge (**C**). Survival rates between vaccinated and unvaccinated animals following SHIV challenges are shown as percentages of animals remaining uninfected after each intrarectal challenge (**D**) and the length they stayed viraemic after confirmation of infection (**E**). (**F**,**G**) Comparison of the levels of SHIV proviral DNA and viral RNA, respectively, between the vaccinated and unvaccinated animals in the PBMC, inguinal (INGU), and mesenteric (MESE) lymph nodes and splenocytes (SPLE) obtained at the experimental endpoint which coincided with 21 weeks after the first SHIV challenge. *ns* = not significant.

Four weeks after the final gp140 Env vaccination, all vaccinated animals, together with the unvaccinated controls were i.r. challenged repeatedly with a low dose of SHIVC109P7. As depicted in Figure 4A,B, both the vaccinated and unvaccinated animals got infected after 1–4 SHIV challenges, with similar median peak viraemia at 1.8 × 10^3^ copies/mL, and 1.6 × 10^4^/mL for the vaccinated and unvaccinated groups, respectively (Figure 4C). The median survival rates between the vaccinated and unvaccinated were also similar at 2 and 2.5 challenges, respectively (Figure 4D) (*p* = 0.065; Gehan–Breslow–Wilcoxon test), with a hazard ratio of 2.08 (Log-rank test). SHIVC109P7 had been used in a previous study where all the inoculated animals rapidly controlled the viraemia to undetectable levels within eight weeks [16]. To evaluate if the vaccines influenced the rapidity of viraemia control, we assessed the length of viraemia from the time of infection to the time the viral loads were consistently below the detection level. Encouragingly, we observed that the length of detectable viraemia in the vaccinated group was shorter than that of the unvaccinated controls (*p* = 0.003; Gehan–Breslow–Wilcoxon test) with a hazard ratio of 5.47 (Log-rank test). Sixty percent (4 of 5) of the vaccinated animals had viraemia for less than 3 weeks while 75% (3 of 4) of the unvaccinated controls had viraemia for >3 weeks (Figure 4E).

We also measured the viral copies in PBMCs, splenocytes, and LMNCs from inguinal and mesenteric lymphoid tissues obtained from SHIV-infected animals at the experimental endpoints. Variable levels of proviral SIV gag DNA were detectable in all the PBMCs and LMNCs from the vaccinated and unvaccinated animals, ranging from 238 to 3,198,678 copies per 10 million cells (Figure 4F). There was no statistical difference between the vaccinated and unvaccinated groups. Viral SIV *gag* RNA was similarly detectable at variable levels in the splenocytes, and LMNCs from the lymph nodes in all animals but at much lower magnitudes, ranging from 5 to 4003 copies per 10 million cells (Figure 4G). The viral RNA was undetected in the PBMCs in 4 of 9 animals infected with SHIV. There was no statistical difference in the number of RNA copies in the blood and tissues between the vaccinated and unvaccinated groups.

### 3.8. Envelope Amino Acid Sequences

**Figure 5:**
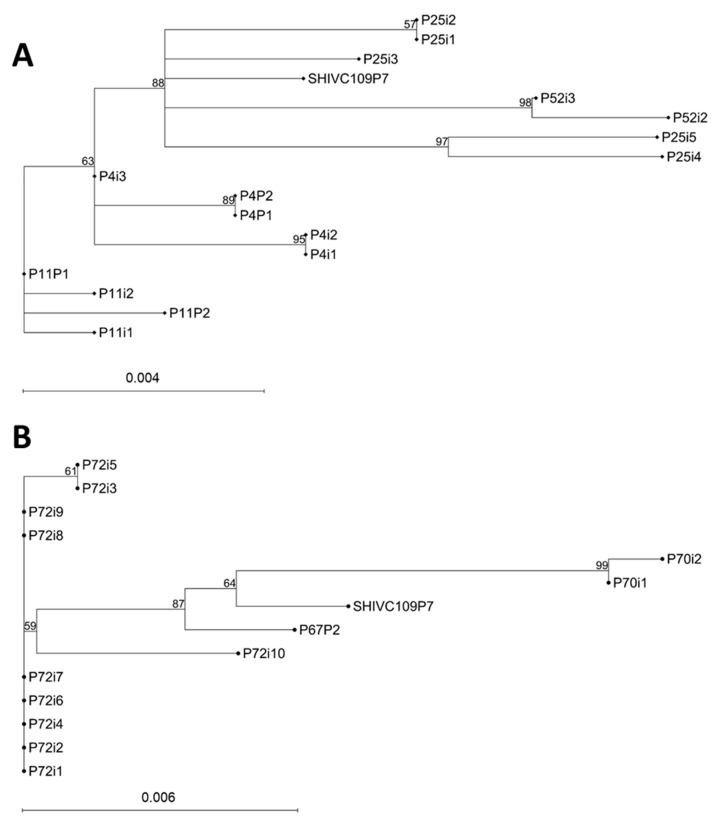
Phylogenetic trees based on the alignment of envelope amino acid sequences from macaques at endpoint. (**A**) Vaccinated animals; (**B**) unvaccinated animals. SHIVC109P87 = consensus sequences of SHIV challenge; i = virus isolated from inguinal lymph node; P = virus isolated from PBMCs. Numbers at the branches denote bootstrap support (1000 iterations). Bootstrap values of ≥50% are shown. The bar at the bottom of the figure denotes genetic distance.

Sequence analysis of viral envelope genes isolated from the PBMCs and inguinal lymph nodes at endpoint were performed. The percentage identity in the vaccinated animals ranged from 98.14 to 100% and the amino acid differences ranged from 0 to 16 (Supplementary Figure S5A). The percentage identity of the envelope sequences isolated from the unvaccinated control group varied from 98.37 to 100% and the amino acid differences ranged from 0 to 14 (Supplementary Figure S5B). Phylogenetic trees were constructed using the Neighbour Joining method (Figure 5). Envelope sequences of viruses isolated from the same macaque were seen to cluster together.

**Figure 6:**
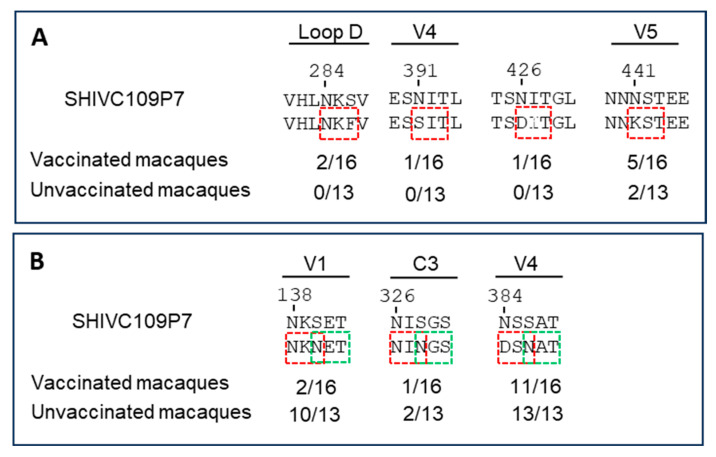
Amino acid sequence comparison of potential N-glycosylation sites (PNGS) in SHIV isolated at end point. (**A**) PNGS that are lost are indicated by red boxes. (**B**) The number of PNGS that have shifted per clones sequenced are indicated below the PNGS. New PNGS are indicated by green boxes.

More potential N-linked glycosylation sites (PNGS) were lost in the vaccinated group (9 in total) than in the unvaccinated group (2 in total) (Figure 6A and Figure S3A). There were also shifts in the PNGS in the V1, C3, and V4 regions (Figure 6B and Figure S3B).

### 3.9. Absolute CD4^+^ T Cells in the Peripheral Blood

To evaluate if viraemia influenced the CD4^+^ T cell counts, we measured CD4^+^ T cells in both the vaccinated and the unvaccinated groups at baseline (pre-challenge) and at several timepoints post-challenge. Both groups had similar counts with medians of 955 (range: 417–1713) and 1111 (range: 501–2062), respectively (Figure S4A,B), which were within the normal ranges for healthy animals. These counts remained generally stable over the 21 weeks following the first SHIV challenge.

## 4. Discussion

Since the evaluation of the SAAVI DNA-C2 and SAAVI MVA-C vaccines in Phase 1 clinical trials [3,5], an impressive level of research efforts in HIV vaccine development has continued in South Africa, with several preclinical [6,7,10,11,12,13,14,29,30,31,32,33,34,35,36] and clinical [37,38] trials being conducted to date. The SAAVI DNA and MVA vaccines elicited robust T cell-mediated responses with limited neutralizing activity, even after incorporation of a gp140 Env protein boost in the clinical trials. A related major Phase 2b/3 trial (Uhambo/HVTN 702) which used gp120 Env to boost canarypox-vectored (ALVAC) primes [39,40,41] was successful in terms of safety evaluation but was prematurely terminated due to lack of efficacy [42,43]. Inducing broadly neutralizing antibodies through vaccination is desirable, although it continues to be a huge challenge [44,45]. However, it has been shown that eliciting T cell responses in addition to nAb is effective in reducing the threshold of nAbs required to confer durable protection [46], further reinforcing the need to include Gag in the envelope-based vaccine designs.

Unfortunately, we were unable to determine the quality of the Env expressed on the surface of the Gag VLPs by assessing the binding of neutralizing and non-neutralizing antibodies due to the lack of resources. The levels of Env trimers incorporated into the VLPs were also not assessed. However, both of these results could have given better insight into the development of B cell responses in vivo [47].

Our research group has incorporated methods that were reported to stabilize the Env trimers [8] and a subtype C mosaic Gag was included in the vaccine formats. We successfully produced DNA and MVA vaccines expressing a SIV Gag and a gp150 HIV-1 envelope protein containing a flexible glycine linker and I559P mutation. These vaccines matched the Gag and Env amino acid sequences of the challenge virus, SHIVC109P7, which contained an HIV subtype C transmitted/founder (T/F) Env ZM109F.PB4 envelope protein [16,17,18,19,20]. We have previously reported a similar strategy in utilizing DNA and MVA vaccines expressing a subtype C mosaic HIV-1 Gag and HIV-1 CAP256SU envelope, which were tested in a rabbit model [10,11,12,13,14]. In the current study, DNA and recombinant MVA vaccines were constructed, which stably expressed SIV Gag and HIV-1 Env (Figure 1) and formed SIV Gag VLPs (Figure 1F). These DNA and MVA vaccines incorporating SIV Gag and Env from SHIVC109P7 as well as the soluble gp140 Env were similar to those expressing the mosaic Gag and Env from the HIV-1 CAP256SU and soluble gp140 Env that we tested in rabbit studies [10,11,12,13,14]. The recent establishment of a SHIV/RM challenge model by our group [16], the only such endeavor in Africa to date, afforded us an opportunity to further evaluate these candidate vaccines in a NHP model where we could assess the potential efficacy of the vaccines.

We tested the immunogenicity of these vaccines using a similar experimental strategy to our rabbit experiments by evaluating the Env antibody mediated as well as the T cell immune responses after two DNA and later two MVA vaccines, the first and the second Env protein vaccines, and at the point of SHIV challenge (pre-challenge). Using an IFN-γ ELISpot method, we observed detectable T cell responses in all the vaccinated animals after the DNA primes and before they received their first MVA booster. This is a noteworthy immunogenicity improvement on the SAAVI DNA-C2 vaccine, which could be attributed to the mammalian expression plasmid pTHPcapR [22] that was used to construct the DNA vaccines. Our SAAVI DNA-C2 vaccine did not elicit detectable IFN-γ ELISpot responses even after 3 DNA immunizations (unpublished). As we had observed before, these responses were significantly boosted by the MVA vaccinations. The capacity of the DNA and MVA to form VLPs might also have improved the observed IFN-γ ELISpot responses as no further boosting effect, in terms of magnitudes, was achieved by subsequent gp140 Env vaccines. In general, each vaccinated animal responded to each vaccine immunogen but at variable magnitudes, possibly reflecting the immunological effect of diverse host factors, such as the major histocompatibility complex alleles [48,49], as we were unable to characterize them in these monkeys. Although the magnitudes declined steadily during the protein vaccinations, all animals had responses by the time of being challenged with SHIV. Gag-specific T cell responses have previously been associated with better viral control in people with HIV and thus, a T cell response to Gag is a desirable outcome for candidate HIV vaccines. We also saw moderate nAb responses against Tier 1A (MW965) and to the autologous Tier 2 (ZM109.5A). No neutralization responses were generated against 6644 (Tier 1B) and ZM109.B4 (Tier 2) pseudoviruses, indicating that induction of vaccine-specific nAbs was limited against these viruses. In contrast, high-level gp140 Env-binding antibodies were elicited after the first protein boost and maintained at similar levels as those elicited by SHIV infection of the unvaccinated animals. However, these high-level binding antibody responses did not correlate with the nAb activity. Due to lack of resources, this study did not implement assays to examine additional T cell and antibody-mediated vaccine-generated immune responses. Thus, future work will explore T cell multi-functionalities beyond IFN-γ ELISpot responses, such as dual IFN-γ/IL-2-producing T cells and their memory subsets. For example, the potential role of vaccine-induced mucosal responses has been attributed to SIV-specific CD4^+^/IL2^+^ and CD8^+^/IFN-γ^+^ T cells [50,51]. Future work should also evaluate vaccine-induced antibody-dependent cellular cytotoxicity (ADCC) and phagocytosis (ADCP) responses to assess the Fc effector functions [52,53].

SHIV challenge models have been widely used in HIV vaccine studies (reviewed in: [54] with repetitive intrarectal doses ranging from as low as 10 TCID_50_ up to as high as 10,000 TCID_50_ (reviewed in [55,56]). In our previous study [16], eight RMs that received i.r. inoculations with undiluted (*n* = 2), a 1:10 (*n* = 2) or a 1:100 (*n* = 4) dilution of the SHIVC109P7 stock were productively infected with a single (undiluted), two (1:10), or 2–3 (1:100) challenges. Thus, we had demonstrated that a low dose of 1:100 dilution of our SHIV stock, corresponding to 6000 TCID_50_, was an optimal low-dose for repetitive i.r. challenges in the current study. Furthermore, Ren et al. [17] had used a similar low dose (5000 TCID_50_) of a related parental virus (SHIVC109P4) stock for intrarectal transmission in Indian-origin RMs. Following repeated i.r. challenges with a 1:100 dilution of SHIVC109P7 in the current study, all vaccinated animals acquired the infection within 1–4 challenges, similar to the unvaccinated controls. The peak median viraemia and the survival rate with respect to the number of challenges resulting in infection were also similar in both groups, indicating that the vaccines did not provide protection against rectal transmission. These parameters were similar to those we obtained in the SHIVC109P7 infection study. However, we observed in the previous study [16] that all the infected animals rapidly controlled the viraemia to undetectable levels within eight weeks. Therefore, we evaluated if the vaccines had an impact on the rapidity of viraemia control and, surprisingly, the length of detectable viraemia in the vaccinated group was significantly shorter than that of the unvaccinated controls. Among the vaccinated animals, 60% of them had viraemia lasting less than 3 weeks compared with 75% of the unvaccinated controls which had viraemia for more than 3 weeks (Figure 4E). Conceivably, the anticipated rapidity in boosting anti-SHIV memory immune responses in the vaccinated animals could have resulted in more potent anti-SHIV Gag and Env immune responses than in the controls. This was supported by the data showing that vaccination resulted in enhancement of SIV Gag and HIV-1 Env IFN-γ ELISpot responses (Figure 3C,D) with the absence of these responses in the control animals coinciding with the time of i.r. challenges. This would have resulted in a lengthy time lag between the start of infection and the onset of effective viral control, reflecting as a longer duration of viraemia. However, the relatively shorter viraemia in the vaccinated animals did not confer any apparent vaccine benefits in terms of less dissemination of SHIV into the crucial viral reservoirs (lymph nodes and spleen) as no statistical difference in the levels of proviral DNA and SIV gag RNA copies could be demonstrated between the vaccinated and unvaccinated groups (Figure 4F,G). It would have been insightful to determine the levels of replication competent viruses in these reservoirs. However, we were unable to do so due to limited resources. Bruner et al. [57] have shown that the majority (>97%) of viral reservoirs had defective viruses during acute HIV-1 infection. Nevertheless, it would have been valuable to evaluate the size of competent proviruses between the vaccinated and unvaccinated animals in our study to understand how vaccine-induced immune responses may have impacted the viral reservoir, albeit the vaccination being non-protective. Future studies could aim to analyze the transcriptional response pre- and post-challenge by applying novel assays such as that described by Bruner et al. [57], which can separately quantify intact and defective proviruses. Recent evidence shows that host immunity exerts selective pressure on reservoir viruses thereby resulting in faster decline of the intact proviral reservoir than defective viruses [58], suggesting a potential beneficial effect of vaccine-enhanced immune responses. However, the elucidation of the exact mechanism responsible for such an effect was beyond the scope of this study. Recent reports of vaccine efficacy against intrarectal SHIV [59,60] have suggested possible mechanisms involving trained innate mucosal immunity such as the stimulation of myeloid cells and expression of platelet factor 4, acting together with antigen-specific T cells. Those studies [59,60] postulated that vaccines can cause alteration of the gut microbiome, which in turn can affect resistance to infection or possibly quicker viral clearance via a rapid production of TNF-α, IL-6, and MIP1α by the innate cells [61]. The antigen-specific T cells may also confer the necessary specificity to guide the trained innate immune cells to the relevant targets.

The sequence analysis data of envelope genes of viruses recovered from the PBMCs and inguinal LMNCs at the experimental endpoint showed similar percentages of amino acid changes in the vaccinated (98.14–100% identity; 0–16 number of amino acids) and unvaccinated (98.37–100% identity; 0–14 number of amino acids) animals compared with the challenge virus (SHIVC109P7). These data, also demonstrated by the phylogenetic trees (Figure 5), confirmed that only modest changes in the envelope sequence occurred after the virus challenge and subsequent infection phase. Except for a few losses of PNGS (mostly the vaccinated animals) and shifting patterns in PNGS in the V1, C3, and V4 (mostly unvaccinated animals) that were noted (Figure 6, Supplementary Figure S3A,B), no gains in PNGS were identified. Taken together, these sequence data suggest that the transmission of challenge virus was not hindered by neutralizing antibodies elicited by the vaccination and the post-infection immune pressure was insufficient to allow the virus to acquire more neutralization resistance. The lack of significant evolution in the env gene during the infection phase could also explain the lack of increased viral replication capacity and depletion of CD4^+^ T cell that were observed (Supplementary Figure S4), which was expected as we had observed in a previous infection study [16]. Env evolution is associated with increased replication and depletion of CD4^+^ T cells [62,63].

## 5. Conclusions

In this study, we demonstrated successful generation of a DNA and an MVA vaccine expressing the SIV Gag and HIV-1 gp150, with the capacity to form SIV Gag VLPs in a similar manner to our candidate HIV-1 vaccines expressing mosaic Gag and CAP256SU Env [10,12,13]. Utilizing our recently established nonhuman primate virus challenge model, we showed that these vaccines elicited strong IFN-γ ELISpot responses, durable gp140 binding antibodies, and moderate autologous Tier 2 antibody responses when used in a heterologous prime-boost combination with soluble gp140 Env in a proof-of-concept study. Although these responses were not protective against a SHIV challenge, they resulted in a shorter length of viraemia, suggesting a potential role of the vaccination in providing enhanced immune capacity to rapidly control viraemia following breakthrough infections. This vaccine may be a candidate therapeutic vaccine for the control of viraemia.

## Data Availability

Data are contained within the article or Supplementary Material.

## Supplementary Materials

The following supporting information can be downloaded at: https://www.mdpi.com/article/10.3390/v17020277/s1, Figure S1: IFN-γ ELISpot responses of individual unvaccinated animals after intrarectal infection with SHIV. Figure S2: Antibody responses following vaccinations. Figure S3A: Comparison of envelopes sequences of the SHIVC109P7 consensus sequence to the isolates derived following challenge in the vaccinated group of macaques. Figure S3B: Comparison of envelopes sequences of the SHIVC109P7 consensus sequence to the isolates derived following challenge in the unvaccinated group of macaques. Figure S4: Absolute CD4^+^ cell counts in the peripheral blood. Figure S5: Pairwise comparisons of envelope sequences showing the percentage identity and number of amino acid differences obtained from macaques at endpoint.
