# Supplementary material for: Heterologous Immunization with Improved HIV-1 Subtype C Vaccines Elicit Autologous Tier 2 Neutralizing Antibodies with Rapid Viral Replication Control After SHIV Challenge
Source: Viruses. 2025 Feb 17;17(2):277. doi: 10.3390/v17020277 (PMC11861162; doi:10.3390/v17020277)
Supplement: Supplementary file 1 [file viruses-17-00277-s001.zip › viruses-3383597-supplementary.pdf]

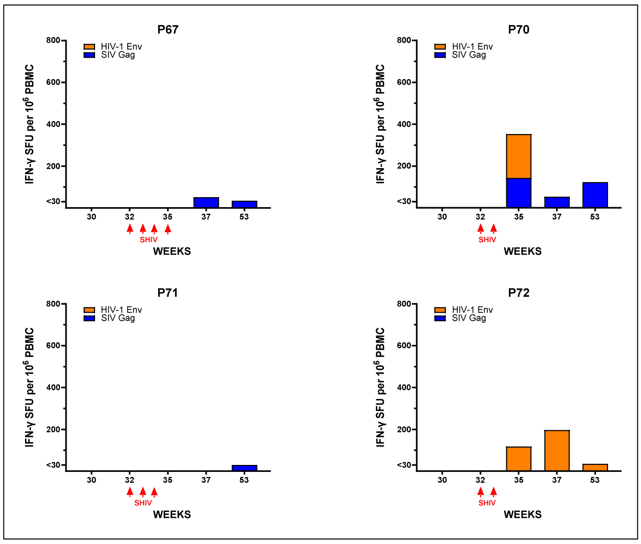

Supplementary Figure S1. Cumulative IFN-γ ELISpot responses of individual unvaccinated animals at pre-challenge (Week 32) and at various timepoints post-challenge intrarectally with SHIV. Each graph represents an individual animal with each bar representing cumulative IFN-γ ELISpot responses to SIV Gag and HIV-1 Env peptide pools measured as spot forming units (SFU) per million PBMC. Magnitudes equal or less than 30 SFU/million PBMC were deemed as negative and assigned zero.

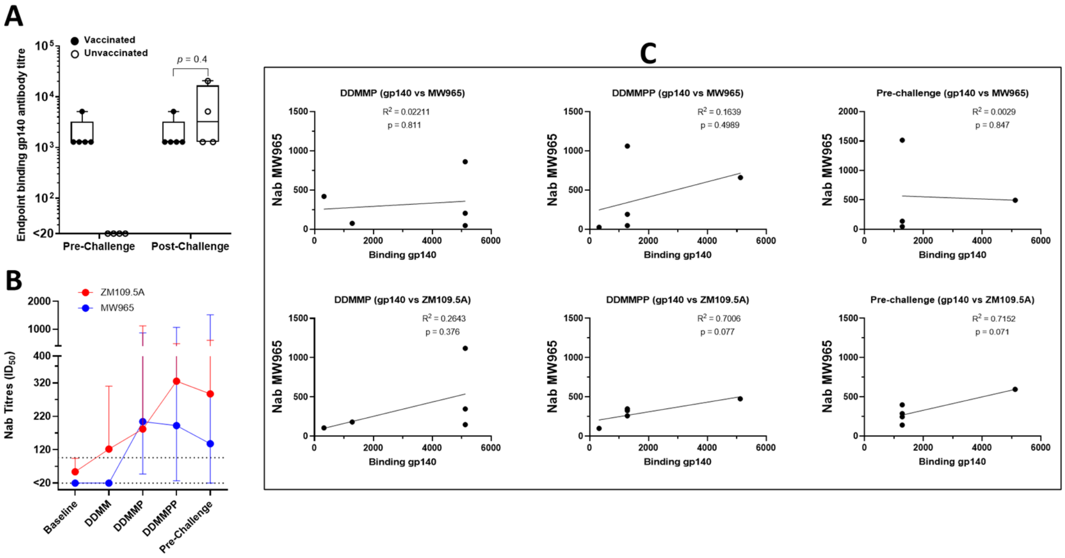

Supplementary Figure S2. Antibody responses following vaccinations. (A) A graph showing Gp140 Env binding antibody titres for vaccinated and unvaccinated animals at pre-challenge (Week 32) and post-challenge (Week 53) timepoints measured by ELISA. Each circle (solid and open for vaccinated and unvaccinated respectively) represents an endpoint titre of an individual animal at the specified timepoint. (B) Line graphs showing median neutralizing antibody titres to MW965 and ZM109.5A pseudovirions at the specified timepoints with vertical lines representing the range. (C) Each graphs show the correlation lines between the gp140 binding endpoint titres and neutralization titres to MW965 and ZM109.5A at the specified timepoints. Values for r-squared (R^2) and *p*-values are given for each graph.

A

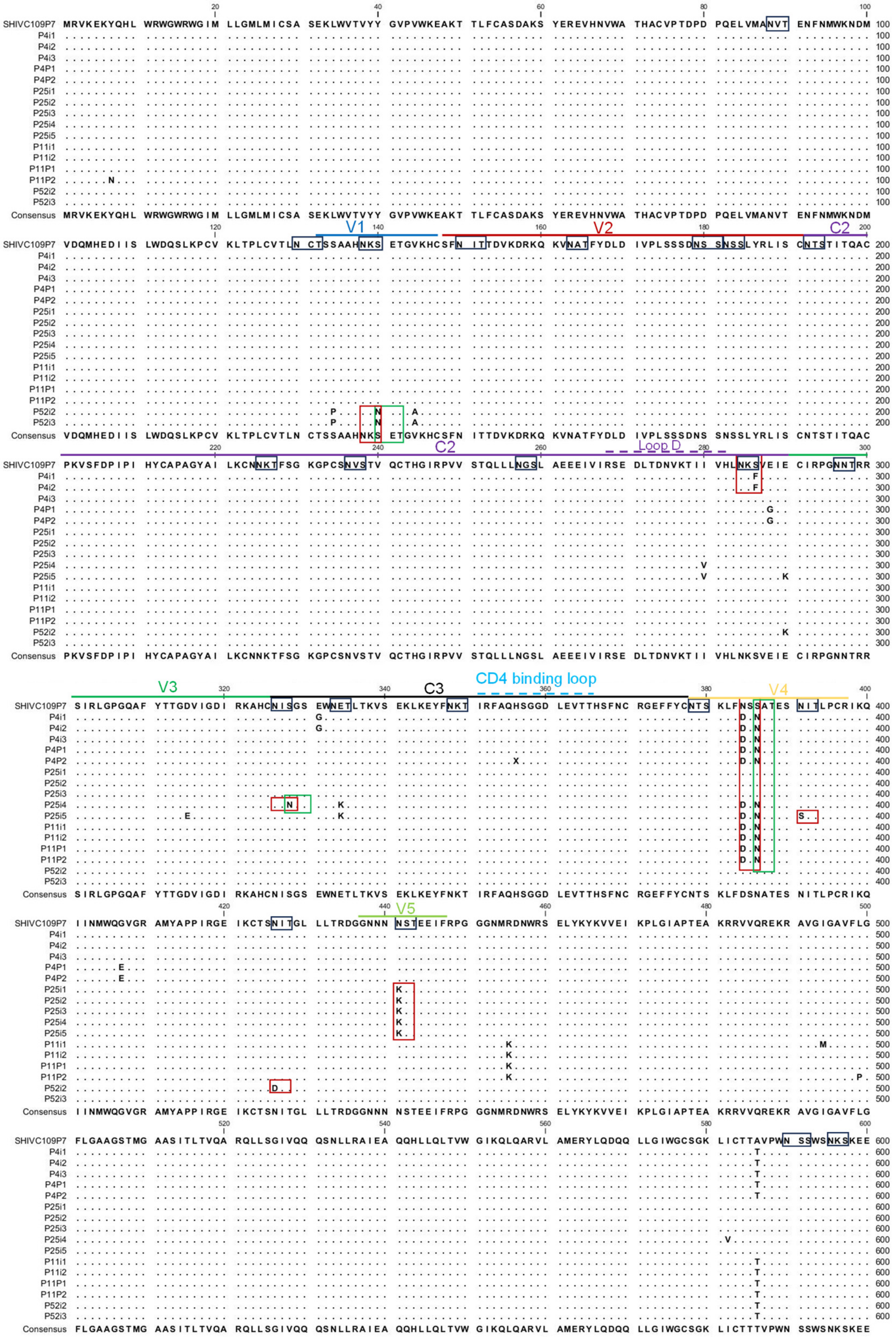

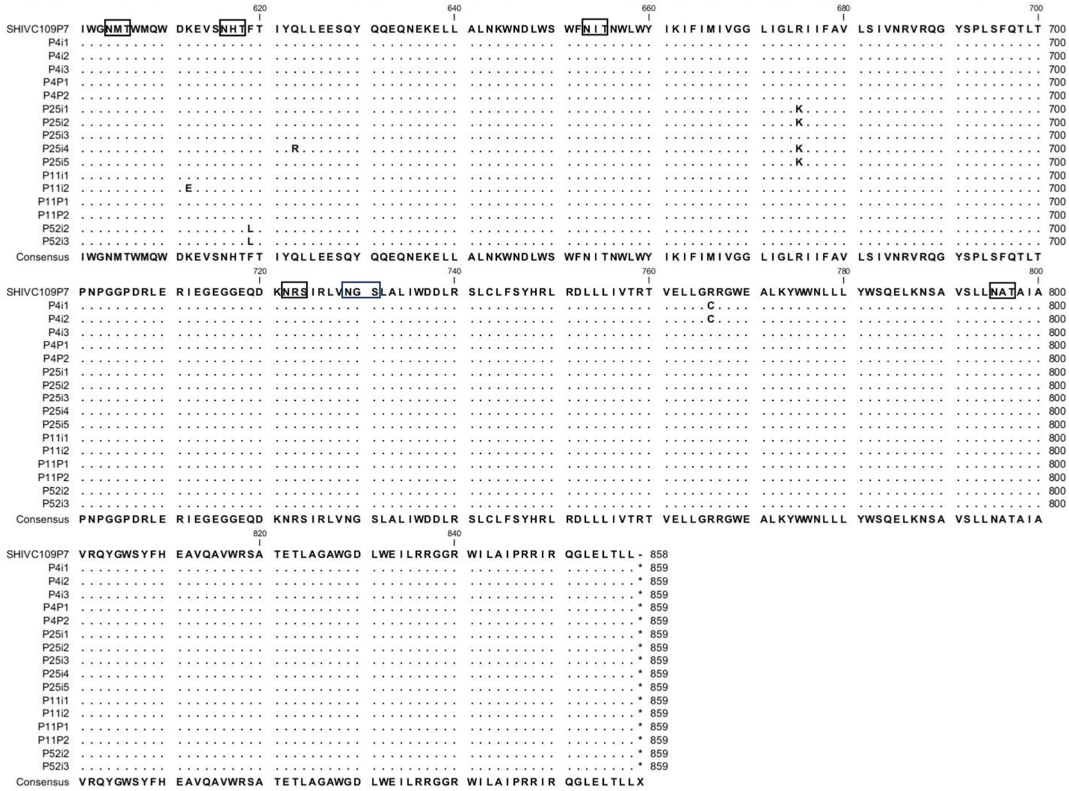

Supplementary Figure S3A. Comparison of envelopes sequences of the SHIVC109P7 consensus sequence to the isolates derived following challenge in the vaccinated group of macaques. i denotes virus isolated from inguinal lymph nodes & P from PBMCs. Potential N-glycosylation sites (PNGS) are bracketed with black boxes, red boxes indicate where glycosylation sites have been lost and green boxes sites where new sites have been gained. Variable regions (V), conserved regions (C) and the amino acids which form part of the CD4 binding site are shown above the sequences.

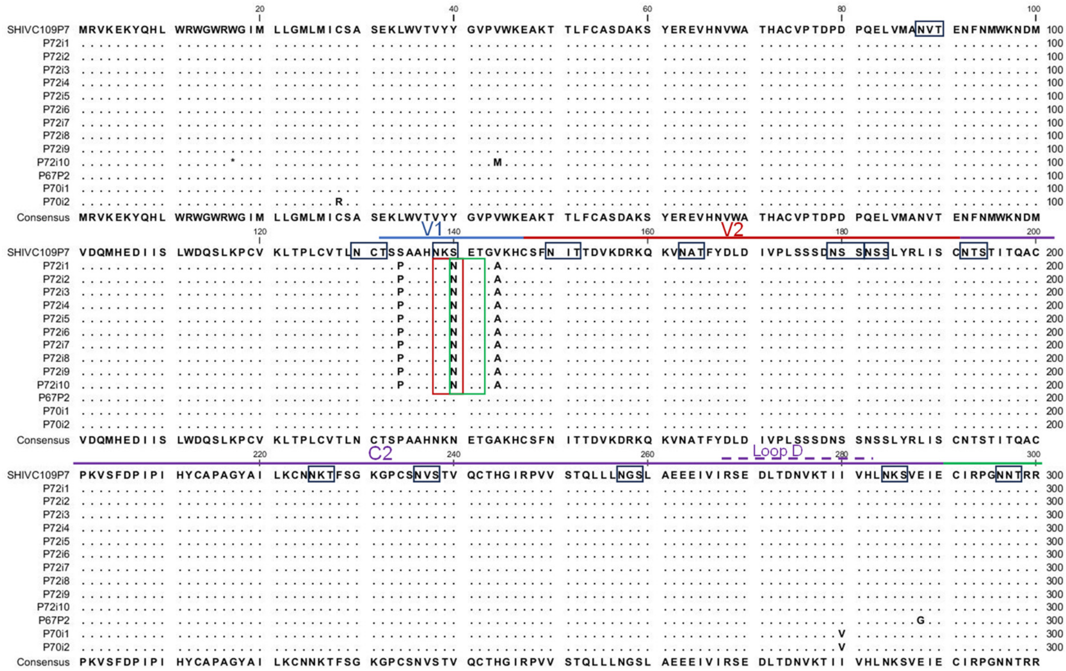

B

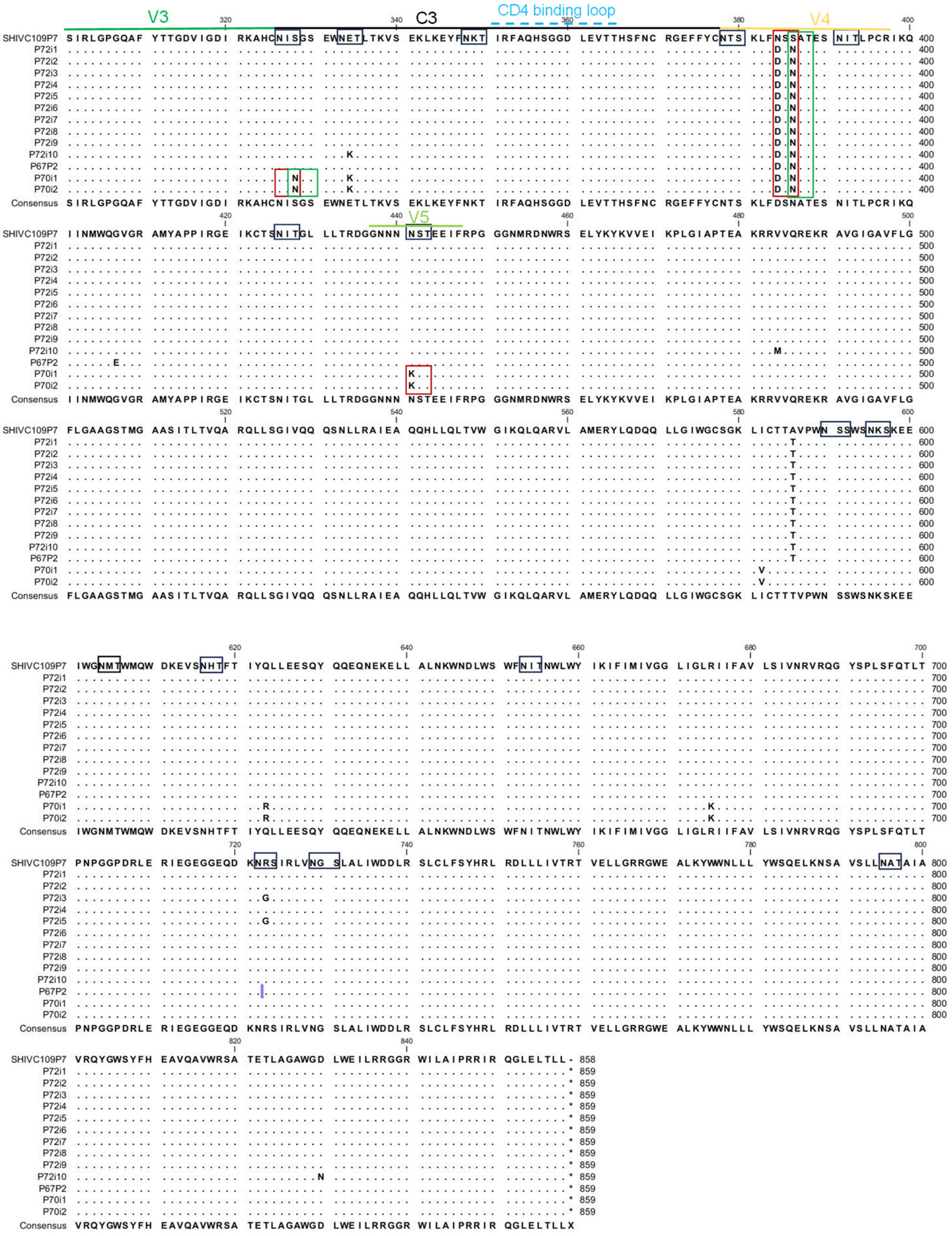

Supplementary Figure S3B. Comparison of envelopes sequences of the SHIVC109P7 consensus sequence to the isolates derived following challenge in the unvaccinated group of macaques. i denotes virus isolated from inguinal lymph nodes & P from PBMCs. Potential N-glycosylation sites (PNGS) are bracketed with black boxes, red boxes indicate where glycosylation sites have been lost and green boxes sites where new sites have been gained. Variable regions (V), conserved regions (C) and the amino acids which form part of the CD4 binding site are shown above the sequences.

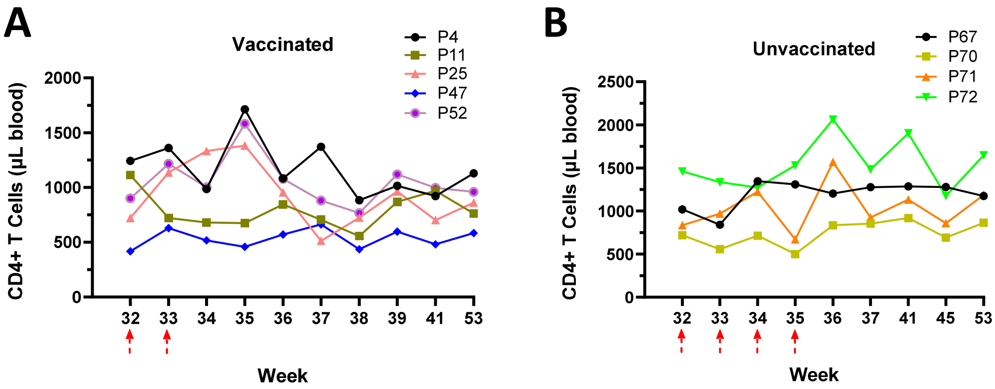

Supplementary Figure S4. Absolute CD4+ cell counts in the peripheral blood of vaccinated (A) and unvaccinated (B) macaques at pre-challenge and at various timepoints post-challenge intrarectally with SHIV. Each line graph represents an individual animal at pre-challenge (Week 32) and post-challenge.

A

		1	2	3	4	5	6	7	8	9	10	11	12	13	14	15	16	17
SHIVC109P7	1		7	7	4	6	6	3	3	2	10	10	6	6	5	7	8	6
P4i1	2	99.19		0	3	5	5	8	8	7	11	11	5	5	4	6	11	9
P4i2	3	99.19	100.00		3	5	5	8	8	7	11	11	5	5	4	6	11	9
P4i3	4	99.53	99.65	99.65		2	2	5	5	4	8	8	2	2	1	3	8	6
P4P1	5	99.30	99.42	99.42	99.77		0	7	7	6	10	10	4	4	3	5	10	8
P4P2	6	99.30	99.42	99.42	99.77	100.00		7	7	6	10	10	4	4	3	5	10	8
P25i1	7	99.65	99.07	99.07	99.42	99.19	99.19		0	1	7	7	7	7	6	8	9	7
P25i2	8	99.65	99.07	99.07	99.42	99.19	99.19	100.00		1	7	7	7	7	6	8	9	7
P25i3	9	99.77	99.19	99.19	99.53	99.30	99.30	99.88	99.88		8	8	6	6	5	7	8	6
P25i4	10	98.84	98.72	98.72	99.07	98.84	98.84	99.19	99.19	99.07		6	10	10	9	11	16	14
P25i5	11	98.84	98.72	98.72	99.07	98.84	98.84	99.19	99.19	99.07	99.30		10	10	9	11	14	14
P11i1	12	99.30	99.42	99.42	99.77	99.53	99.53	99.19	99.19	99.30	98.84	98.84		2	1	3	10	8
P11i2	13	99.30	99.42	99.42	99.77	99.53	99.53	99.19	99.19	99.30	98.84	98.84	99.77		1	3	10	8
P11P1	14	99.42	99.53	99.53	99.88	99.65	99.65	99.30	99.30	99.42	98.95	98.95	99.88	99.88		2	9	7
P11P2	15	99.19	99.30	99.30	99.65	99.42	99.42	99.07	99.07	99.19	98.72	98.72	99.65	99.65	99.77		11	9
P52i2	16	99.07	98.72	98.72	99.07	98.84	98.84	98.95	98.95	99.07	98.14	98.37	98.84	98.84	98.95	98.72		2
P52i3	17	99.30	98.95	98.95	99.30	99.07	99.07	99.19	99.19	99.30	98.37	98.37	99.07	99.07	99.19	98.95	99.77	

B

		1	2	3	4	5	6	7	8	9	10	11	12	13	14
SHIVC109consensus	1		7	7	8	7	8	7	7	7	7	11	6	10	11
P72i1	2	99.19		0	1	0	1	0	0	0	0	4	5	11	12
P72i2	3	99.19	100.00		1	0	1	0	0	0	0	4	5	11	12
P72i3	4	99.07	99.88	99.88		1	0	1	1	1	1	5	6	12	13
P72i4	5	99.19	100.00	100.00	99.88		1	0	0	0	0	4	5	11	12
P72i5	6	99.07	99.88	99.88	100.00	99.88		1	1	1	1	5	6	12	13
P72i6	7	99.19	100.00	100.00	99.88	100.00	99.88		0	0	0	4	5	11	12
P72i7	8	99.19	100.00	100.00	99.88	100.00	99.88	100.00		0	0	4	5	11	12
P72i8	9	99.19	100.00	100.00	99.88	100.00	99.88	100.00	100.00		0	4	5	11	12
P72i9	10	99.19	100.00	100.00	99.88	100.00	99.88	100.00	100.00	100.00		4	5	11	12
P72i10	11	98.72	99.53	99.53	99.42	99.53	99.42	99.53	99.53	99.53	99.53		9	13	14
P67P2	12	99.30	99.42	99.42	99.30	99.42	99.30	99.42	99.42	99.42	99.42	98.95		10	11
P70i1	13	98.84	98.72	98.72	98.60	98.72	98.60	98.72	98.72	98.72	98.72	98.49	98.84		1
P70i2	14	98.72	98.60	98.60	98.49	98.60	98.49	98.60	98.60	98.60	98.60	98.37	98.72	99.88	

Supplementary Figure S5. Pairwise comparisons of envelope sequences showing the percentage identity and number of amino acid differences obtained from macaques at endpoint. (A) Vaccinated animals. (B) Unvaccinated animals. SHIVC109P7 = consensus sequence of SHIV challenge virus. i = virus isolated from inguinal lymph nodes; P = virus isolated from PBMCs.
